# Estimation of Vaccine Efficacy and Critical Vaccination Coverage in Partially Observed Outbreaks

**DOI:** 10.1371/journal.pcbi.1003061

**Published:** 2013-05-02

**Authors:** Michiel van Boven, Wilhelmina L. M. Ruijs, Jacco Wallinga, Philip D. O'Neill, Susan Hahné

**Affiliations:** 1 Centre for Infectious Disease Control, National Institute for Public Health and the Environment, Bilthoven, The Netherlands; 2 Academic Collaborative Centre AMPHI, Department of Primary and Community Care, Radboud University Nijmegen Medical Centre, Nijmegen, The Netherlands; 3 School of Mathematical Sciences, University of Nottingham, Nottingham, United Kingdom; Imperial College London, United Kingdom

## Abstract

Classical approaches to estimate vaccine efficacy are based on the assumption that a person's risk of infection does not depend on the infection status of others. This assumption is untenable for infectious disease data where such dependencies abound. We present a novel approach to estimating vaccine efficacy in a Bayesian framework using disease transmission models. The methodology is applied to outbreaks of mumps in primary schools in the Netherlands. The total study population consisted of 2,493 children in ten primary schools, of which 510 (20%) were known to have been infected, and 832 (33%) had unknown infection status. The apparent vaccination coverage ranged from 12% to 93%, and the apparent infection attack rate varied from 1% to 76%. Our analyses show that vaccination reduces the probability of infection per contact substantially but not perfectly (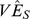 = 0.933; 95CrI: 0.908–0.954). Mumps virus appears to be moderately transmissible in the school setting, with each case yielding an estimated 2.5 secondary cases in an unvaccinated population (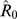 = 2.49; 95%CrI: 2.36–2.63), resulting in moderate estimates of the critical vaccination coverage (64.2%; 95%CrI: 61.7–66.7%). The indirect benefits of vaccination are highest in populations with vaccination coverage just below the critical vaccination coverage. In these populations, it is estimated that almost two infections can be prevented per vaccination. We discuss the implications for the optimal control of mumps in heterogeneously vaccinated populations.

## Author Summary

Less than two decades ago, it was generally believed that in developed countries infectious diseases such as measles, mumps, and pertussis were under firm control via vaccination. Nowadays, it is increasingly recognized that this picture has been overly optimistic. A central question is whether recurrent disease outbreaks are caused by vaccination coverage having dropped below safe levels, or by vaccines having become less effective. To answer this question, the authors study outbreaks of mumps in primary schools in the Netherlands. Using disease transmission models, the authors estimate vaccine efficacy and the critical vaccination coverage needed to prevent large outbreaks. The analyses show that the vaccine has been highly effective in preventing infection, but that vaccination coverage has been insufficient in some schools. The authors argue that catch-up vaccination campaigns aimed at populations with intermediate vaccination coverage will be most efficient, as these would maximize the (direct and indirect) benefits of vaccination.

## Introduction

Mass vaccination programs for childhood diseases have been highly successful in reducing the incidence and public health impact of the targeted diseases. Nevertheless, with the exception of smallpox, eradication has not been achieved, and outbreaks continue to occur even in highly vaccinated populations [1]–[4]. A prominent example is that of mumps, which has re-emerged in the past decade in highly vaccinated populations throughout the world [5]–[7]. The question arises as to whether this re-emergence is due to current vaccines becoming less effective, or to reduced vaccine coverage which allows the virus to spread in partially vaccinated populations [8].

In the Netherlands, large outbreaks of mumps genotypes D and G have occurred in recent years [9]–[11]. Since 1987, a combined MMR (measles-mumps-rubella) vaccine containing live attenuated virus is routinely given at 14 months and 9 years of age. Vaccination coverage has been high ever since introduction of the vaccine in 1987 (90–95%). Nevertheless, there are municipalities in which vaccination coverage is substantially lower [12], [13].

To determine whether the outbreaks of mumps are the result of low vaccination coverage or insufficient protection conferred by the vaccine, we estimate vaccine efficacy using outbreak data from ten primary schools in the Netherlands [9], [11]. The total number of children included in our study is 2,493, of whom 510 had a reported mumps infection. Vaccination coverage in these schools ranged from 12%–93%, and infection attack rates ranged from 4% to 76%, with highest attack rates occurring in schools with the lowest vaccination coverage and lowest attack rates in schools with high vaccination coverage (Table 1). Notably, the attack rates in unvaccinated individuals varied from more than 80% in schools with low vaccination coverage (<15%) to lower than 25% in schools with high vaccination coverage (≥75%), indicating substantial differences in the infection pressure between schools.

**Table 1:** Summary statistics of the study population.

	number of persons	number infected	vaccination coverage	attack rate in unvaccinated persons		attack rate in vaccinated persons		overall attack rate
all schools	2493	510–1342	0.62*	0.68	(485/709)	0.03	(25/952)	0.31
school 1	432	205–369	0.12	0.86	(204/237)	0.03	(1/31)	0.76
school 2	338	135–289	0.13	0.82	(131/160)	0.17	(4/24)	0.73
school 3	259	68–159	0.42	0.72	(68/94)	0	(0/74)	0.40
school 4	184	40–70	0.54	0.53	(37/70)	0.04	(3/84)	0.26
school 5	130	13–33	0.75	0.46	(13/28)	0	(0/82)	0.12
school 6	263	28–171	0.76	0.70	(19/27)	0.10	(9/93)	0.23
school 7	194	6–43	0.78	0.19	(6/31)	0	(0/126)	0.04
school 8	227	3–27	0.79	0.05	(2/41)	0.01	(1/162)	0.01
school 9	258	6–119	0.93	0.18	(2/11)	0.03	(4/134)	0.04
school 10	208	6–62	0.93	0.30	(3/10)	0.02	(3/142)	0.04

The column ‘number infected’ shows the possible range of actual infections, ranging from the number known to be infected to the sum of this number and the number of persons with unknown infection status. Vaccination coverages and attack rates are calculated using persons with known vaccination status (vaccination coverage), and known vaccination and infection status (attack rates). See Tables S1, S2 for the complete data.

* : averaged over schools.

Classical methods to estimate vaccine efficacy from outbreak data compare the infection attack rates in the vaccinated versus unvaccinated groups (i.e. the cohort method) [14], [15]. This method, however, has significant drawbacks. First, it is not straightforward to take account of missing data on vaccination and infection status. This is unfortunate as outbreak data are almost never complete, and judicious choices will have to be made to avoid introducing systematic bias in the parameter estimates. Even more importantly, the cohort method fails to acknowledge that the probability of infection of an individual is dependent on the number of infections in the population, i.e. on the infection status of others.

To take account of the dependencies between individuals that arise naturally in infectious disease outbreaks we base the statistical analyses on a Bayesian inferential framework using infectious disease transmission models. In this framework, missing vaccination and infection information is imputed in a consistent manner, thereby making efficient use of the available information, and enabling precise estimation of vaccine efficacy and the critical vaccination coverage needed to prevent epidemic outbreaks [16], [17]. The basis of our statistical analyses is the contact process that specifies how often and with which person-types each person makes infectious contacts, i.e. contacts that are sufficient for transmission if the sender is infected and the receiver as yet uninfected [18]–[20]. The contact process specifies a directed graph, of which the connected component with the initial infective as the root determines which individuals are ultimately infected. Estimation of the epidemiological parameters (basic reproduction number, vaccine efficacy) is based on the likelihood of directed graphs that are compatible with the data.

The analyses reveal that mumps vaccine effectively prevents infection, and that herd immunity against mumps is achieved with moderate vaccination coverages. We argue that resource-limited catch-up vaccination efforts should be focused at communities with intermediate vaccination coverages, thereby maximizing both the direct and indirect benefits of vaccination.

## Results

### Vaccine efficacy and the critical vaccination coverage

**Figure 1:**
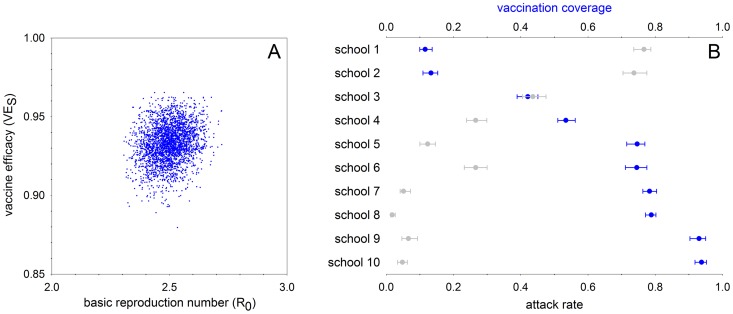
Posterior distributions of the basic reproduction number and vaccine efficacy (A) and vaccination coverages and attack rates (B) when assuming common parameters across schools (baseline scenario). The median of the basic reproduction number is 2.49 (95% CrI: 2.36–2.63) and the median of vaccine efficacy is 0.933 (95CrI: 0.908–0.954). The estimated critical vaccination coverage is 0.642 (95% CrI: 0.617–0.666).

Our baseline scenario assumes a common transmissibility and vaccine efficacy across schools. The analysis indicates that mumps is moderately transmissible (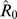 = 2.49; 95%CrI: 2.36–2.63), and that the vaccine reduces the probability of transmission by more than 90% per contact that would have resulted in transmission to an unvaccinated person (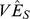 = 0.933; 95CrI: 0.908–0.954)(Figure 1). The differences between the apparent and estimated vaccination coverages and attack rates are small (<2% and <5%, respectively; Tables 1–2).

**Table 2:** Estimates of vaccination coverage and attack rate per school when assuming common epidemiological parameters across schools (baseline scenario).

	unvaccinated		vaccinated		vaccination coverage	attack rate
	total	infected	total	infected		
school 1	382 (372–389)	327 (315–337)	50 (43–59)	3 (1–6)	0.12 (0.10–0.14)	0.77 (0.74–0.79)
school 2	293 (286–301)	243 (232–256)	45 (37–52)	6 (4–11)	0.13 (0.11–0.15)	0.74 (0.70–0.78)
school 3	150 (141–158)	110 (101–119)	109 (101–117)	3 (0–8)	0.42 (0.39–0.45)	0.44 (0.41–0.47)
school 4	84 (79–90)	45 (41–50)	100 (94–104)	4 (3–6)	0.53 (0.51–0.56)	0.27 (0.24–0.30)
school 5	33 (30–37)	15 (13–18)	97 (93–100)	0 (0–2)	0.75 (0.72–0.77)	0.12 (0.10–0.15)
school 6	67 (59–76)	50 (42–59)	196 (187–204)	20 (14–27)	0.75 (0.71–0.78)	0.27 (0.23–0.30)
school 7	42 (38–46)	10 (7–13)	152 (148–156)	0 (0–3)	0.78 (0.76–0.80)	0.05 (0.04–0.07)
school 8	48 (45–52)	3 (2–5)	179 (175–182)	1 (1–2)	0.79 (0.77–0.80)	0.02 (0.01–0.03)
school 9	18 (13–25)	6 (3–11)	240 (233–245)	11 (7–16)	0.93 (0.90–0.95)	0.07 (0.05–0.09)
school 10	13 (10–17)	4 (3–7)	195 (191–198)	5 (3–8)	0.94 (0.92–0.95)	0.05 (0.03–0.06)

Estimates are represented by posterior medians with 95% credible intervals.

**Figure 2:**
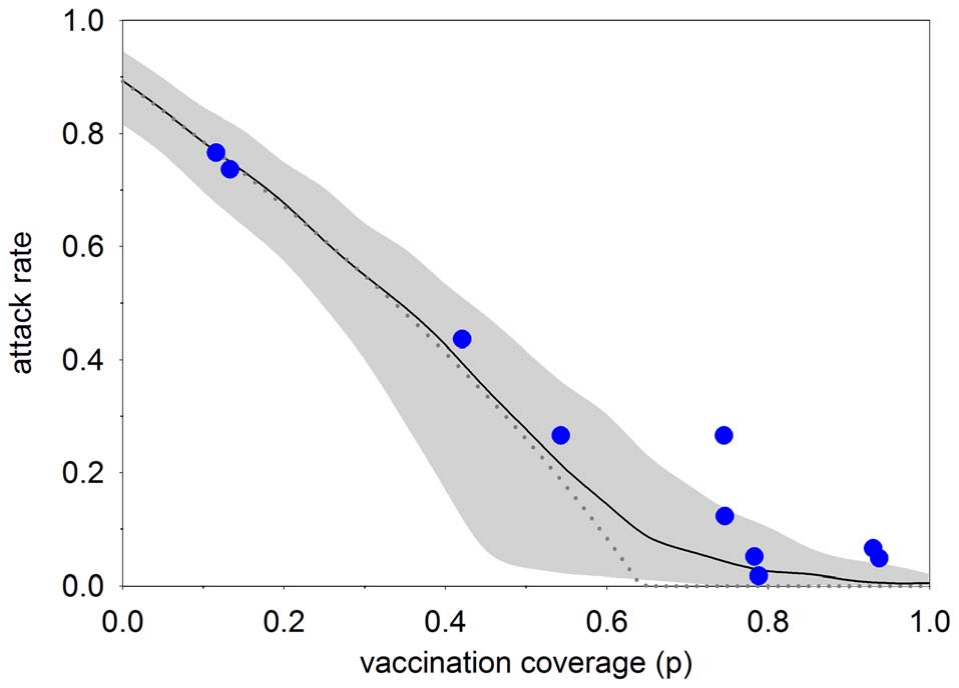
Relation between vaccination coverage and overall attack rate in the baseline scenario. The figure shows the medians of the posterior vaccination coverages versus posterior attack rates in the ten schools (blue dots), the deterministic final size attack rate using the posterior medians of the basic reproduction number and vaccine efficacy (dotted line), and the results of simulations in populations of size 200 using samples from the posterior distributions of the basic reproduction number and vaccine efficacy (black line: median; grey area: 2.5%–97.5% percentiles). See text for details.

We use estimates of transmissibility and vaccine efficacy to obtain estimates of the critical vaccination coverage. The analyses yield an estimated critical vaccination coverage of 0.642 (95%CrI: 0.617–0.666), indicating that herd immunity in the school setting can be obtained with moderate vaccination coverages. Estimates of transmissibility and vaccine efficacy are used to obtain an estimate of the number of infections prevented per vaccination. This number is highest for vaccination coverages just below the critical vaccination coverage, as at these values the slope of attack rate versus vaccination coverage is steepest (Figure 2). The number of infections prevented per vaccination near the threshold coverage is well approximated (using a Taylor series expansion) by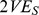. Hence, it is expected that the (direct and indirect) benefits of vaccination are such that 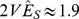 infections can be prevented per vaccination if the initial vaccination coverage is just below the threshold value, which is estimated by 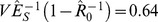.

### Schools with low versus high vaccination coverage

Schools in our study population span a large range of possible vaccination coverages, and it is of interest to evaluate the consistency of the estimates of vaccine efficacy and pathogen transmissibility. Figure 2 shows the relation between vaccination coverage and infection attack rate in the ten schools, together with the theoretical relation between vaccination coverage and attack rate in a large population, and simulations of a finite population. Overall, the correspondence between the observed and simulated data is excellent for schools with low vaccination coverage and high attack rates, while there is a tendency for higher attack rates than expected in schools with high vaccination coverage and a small number of infections.

**Figure 3:**
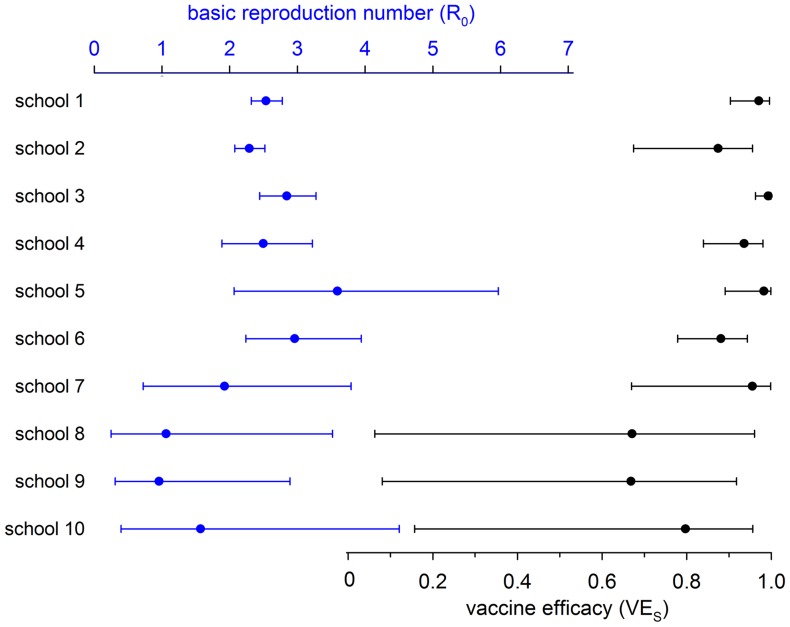
Estimated reproduction numbers (top, blue dots) and vaccine efficacies (bottom, gray dots) per school, with associated 95% credible intervals (cf. Table 3). Note that estimated vaccine efficacy is consistently high in schools with high exposure (schools 1–6), but cannot be estimated with any precision in schools with low exposure (schools 8–10).

To investigate the information contained in the data by school we perform analyses in which each school is equipped with its own transmissibility and vaccine efficacy. It appears that precise estimates of transmissibility and vaccine efficacy can be obtained in schools with high attack rates (schools 1–4), but not in schools with only a handful of infections (schools 7–10). In fact, in schools with less than 10 confirmed infections credible intervals of the reproduction number range from well below 1 to more than 3, while vaccine efficacy estimates can range from less than 0.20 (schools 8–10) to almost 1 (schools 7–10; Table 3, Figure 3). Further, the analyses show that in schools with high attack rates (schools 1–4) the parameter estimates are quite close to those of the baseline scenario, indicating that estimates of transmissibility and vaccine efficacy in the baseline scenario are dominated by schools with large numbers of infections and low vaccination coverages.

**Table 3:** Overview of the analyses per school (cf. Figure 3).

	basic reproduction number 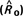	vaccine efficacy 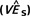	critical vaccination coverage 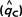
school 1	2.5 (2.3–2.7)	0.97 (0.91–1.0)	0.63 (0.59–0.67)
school 2	2.3 (2.1–2.5)	0.87 (0.67–0.96)	0.65 (0.57–0.80)
school 3	2.8 (2.4–3.3)	0.99 (0.96–1.0)	0.66 (0.60–0.70)
school 4	2.5 (1.9–3.2)	0.94 (0.84–0.98)	0.64 (0.51–0.74)
school 5	3.6 (2.1–6.0)	0.98 (0.89–1.0)	0.74 (0.54–0.85)
school 6	3.0 (2.2–3.9)	0.88 (0.78–0.94)	0.76 (0.66–0.83)
school 7	1.9 (0.73–3.8)	0.95 (0.67–1.0)	0.51 (0–0.77)
school 8	1.1 (0.25–3.5)	0.67 (0.06–0.96)	0.10 (0–1)
school 9	0.96 (0.31–2.9)	0.67 (0.08–0.92)	0 (0–0.74)
school 10	1.6 (0.40–4.5)	0.80 (0.16–0.96)	0.46 (0–0.85)

The table shows the estimates of the basic reproduction number, vaccine efficacy, and critical vaccination coverage. Estimates are represented by posterior medians with 95% credible intervals.

### Estimation of vaccine efficacy by the cohort method

In comparison with our estimates of vaccine efficacy as the reduction in the probability of infection (Table 3), estimates of vaccine efficacy by the cohort method tend to be somewhat lower in schools with low vaccination coverage and high infection attack rates (schools 1–4; Table 4, Table S3). Moreover, in these schools credible intervals tend to be slightly broader when using the cohort method. The most conspicuous difference, however, is that in populations with high vaccination coverage (schools 7–10), vaccine efficacy is sometimes estimated with fair precision when using the cohort method, even though the number of infections is very small (≤6).

**Table 4:** Estimates of vaccine efficacy by the cohort method, i.e. as 1 minus the relative risk of infection in vaccinated versus unvaccinated persons (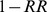).

	vaccine efficacy 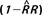
school 1	0.93 (0.81–0.99)
school 2	0.76 (0.56–0.92)
school 3	0.98 (0.93–1.0)
school 4	0.91 (0.80–0.98)
school 5	0.97 (0.90–1.0)
school 6	0.84 (0.73–0.93)
school 7	0.96 (0.83–1.0)
school 8	0.75 (0.0–0.98)
school 9	0.78 (0.25–0.96)
school 10	0.90 (0.68–0.98)

Estimates are represented by posterior medians with 95% credible intervals. See text for details.

## Discussion

Our analyses have shown that mumps is moderately transmissible in the setting of primary schools, and that the vaccine used in these populations is highly effective in preventing infection. These results are largely in line with earlier studies [5], [6], but contrast with a recent study that suggested that outbreaks of mumps in populations with large-scale vaccination programs may be due to the vaccine having become less effective in preventing infection [4]. The younger average age of our study population and the fact that these outbreaks have been caused by viruses of different genotypes (genotype D versus genotype G) may help explain these contrasting findings. Since genotype D viruses are genetically distant from the current vaccine virus (Jeryl Lynn strain, genotype A) our results indicate that the Jeryl Lynn-based vaccine is highly effective in curbing transmission to vaccinated persons, even if genetic differences between the vaccine and outbreaks strains are substantial [8].

Estimates of the transmissbility of mumps are most precise in schools 1–4, i.e. in schools with low vaccination coverage and large numbers of infections. In these schools, the basic reproduction number is estimated at 2.5, 2.3, 2.8, and 2.5, with credible intervals ranging from 1.9 to 3.2. Vaccine efficacy, on the other hand, is estimated most precisely in schools 1, 3, 4, and 5 (Table 3, Figure 3). In these schools, estimates of vaccine efficacy are 0.97, 0.99, 0.94, and 0.98, with credible intervals ranging from 0.84 to 1. These schools have low vaccination coverage and high levels of exposure (i.e. high attack rates) but still more than 30 vaccinated persons. In school 2 the exposure level has been high but the number of vaccinated persons is too small for precise estimation of vaccine efficacy. In schools with high coverage, vaccine efficacy cannot be estimated with any precision, as in these schools it is uncertain whether escape from infection is caused by the vaccine or by a lack of exposure.

The schools included in this study differ greatly with respect to vaccination coverages (range: 12%–93%) and infection attack rates (range: 4%–76%). Nevertheless, estimates of vaccine efficacy are remarkably consistent across schools (Table 3, Figure 3). In fact, only in schools with just a handful of infections (≤6) (schools 8–10) does the estimated vaccine efficacy drop below 0.88. In these schools, credible intervals of vaccine efficacy are wide, and estimates are less determined by the information contained in the data than by the prior distribution of vaccine efficacy. This is also the reason that estimates of vaccine efficacy in the baseline scenario are dominated by schools with low vaccination coverages and high attack rates, as these schools contain much more information than schools with high vaccination coverages and low infection attack rates (Figure 2).

Schools in our study were included based on confirmed mumps infections. It is therefore possible that large outbreaks are more likely to be detected and included than small outbreaks. In other words, it is conceivable that the inclusion process systematically favours inclusion of schools with uncharacteristically high attack rates, thereby leading to selection bias. For schools with low vaccination coverage (and high attack rates) this is arguably not a problem as variation in outbreak sizes is expected to be minor, given the sizes of the schools included (Figure 2). For schools with high vaccination coverage, however, selection bias may well have played a role, and may explain the relatively high attack rates in some of these schools (school 6 and to a lesser extend schools 9–10) (Figure 2). Fortunately, one could argue that our statistical methodology provides a natural weighting of schools, in which schools with small number of infections have lower weight than schools with high number of infections. If specific details were available on the inclusion process, one could envisage extension of the analyses in which the selection process is modelled explicitly. This, however, would introduce more model options, additional parameters to be estimated, and would certainly lead to a more complicated analysis.

We have assumed throughout that infections outside the school played a marginal role. Again, this assumption is probably less problematic in schools with low vaccination coverage and high infection attack rates than in schools with high vaccination coverage and lower attack rates, as variation in the expected number of infections is expected to be small in schools with low vaccination coverage. Moreover, there was no sustained community transmission during the study period, suggesting that the impact of infection outside the schools may have been small. Nevertheless, it would be interesting to extend the current analyses, e.g., along the lines of [21], [22] by inclusion of other major transmission settings.

Classical estimates of mumps transmissibility have been based on the mean age at infection in the pre-vaccination era ([23] and references therein), or on seroprevalence data from the pre-vaccination era [24], [25]. These analyses yielded estimates of the basic reproduction number in fully unvaccinated populations that are substantially higher (∼7–20) than our estimates (∼2–3). It should be noted that these population-based estimates cannot directly be translated to our school-based estimates. Still, should those early estimates be indicative of the current transmissibility of mumps at the population level, then not only are schools an important transmission route but other settings also have the potential to contribute significantly to overall transmission. Again, to assess the contribution of different settings to the overall transmission dynamics, it would be desirable to extend the current studies beyond the school setting, by including household information and, in the specific case of this study, information on the churches attended by the participants [9]. This, however, is only possible if detailed information were available on these settings, not only with respect to their composition but also with respect to vaccination and infection status of a sizeable part of the population.

Vaccine efficacy and transmissibility together determine the critical vaccination coverage needed to prevent epidemic outbreaks. In our study, estimates of the critical vaccination coverage are 64% (95%CrI: 62%–67%) in the baseline scenario, and range from 63% (95%CrI: 58%–68%; school 1) to 76% (95%CrI: 66%–83%; school 6) in schools with more than 10 confirmed infections (schools 1–6). This indicates that the critical vaccination coverage does not need to be as high as suggested by early population-based estimates, which are in the range of 86%–95%.

In none of the analyses presented here have we made a distinction between children who had been vaccinated once and those that had been vaccinated twice. This was done because preliminary analyses and previous results [9], [11] could not find any evidence for differences in vaccine efficacy between the two groups. In view of the data this is not unexpected, as the total number of infections in vaccinated children was small, and as attack rates in the two subpopulations were identical (15 infections among the 582 children who had been vaccinated once; 10 infections among the 370 who had been vaccinated twice). The fact that attack rates were identical is somewhat surprising, as one could have expected more infections in the group that had been vaccinated only once, more than five years ago. For completeness, we have presented the full data in Table S2.

Further, in our analyses we assume that the vaccine works by reducing the probability of transmission (i.e. we assume a leaky vaccine), rather than by providing all-or-nothing immunity. This was done for simplicity, and since the current data do not allow us to distinguish between the different workings of the vaccine. If additional data was available, e.g., on the pre-outbreak antibody titres, one could consider extension of the method by using pre-outbreak antibody titres as an indicator for the ‘level of immunity’, and use this indicator to estimate how the level of pre-existing immunity relates to the probability of infection. In most situations, however, such information will be hard to get, as this would necessitate a large prospective study.

Our definition of vaccine efficacy has a clear-cut biological interpretation (reduction of the probability of infection per contact). This makes it possible to meaningfully average over populations with varying vaccination coverages and exposure levels, and also to extrapolate beyond the study population. This contrasts with traditional estimates of vaccine efficacy that are based on a comparison of attack rates in vaccinated and unvaccinated individuals (the cohort method), or that simply use the vaccination status of the infected individuals together with the population vaccination coverage (the screening method) [14], [26]. Vaccine efficacy estimated by these methods lack a clear biological interpretation, and in essence assumes that a person's risk of infection is independent of whether or not others in the population are infected. This makes interpretation of the estimates problematic, and forbids estimation of the critical vaccination coverage [15], [27]–[29].

Even though our definition of vaccine efficacy differs fundamentally from vaccine efficacy measured by the cohort method, the results are quantitatively in fair agreement with traditional estimates, especially in populations with low vaccination coverage and large number of infections (Table 3 versus Table 4 and Table S3). In schools with high vaccination coverage and small numbers of infections the reverse tends to be true, and estimates of vaccine efficacy generally are both higher and more precise when using the cohort method. For instance, in school 10 there are 6 confirmed infections, and vaccine efficacy is poorly estimated in our analysis (95%CrI: 0.16–0.96) but with fair precision by the cohort method (95%CrI: 0.68–0.98). This is arguably an artefact of the latter method's assumption that all 139 uninfected vaccinated persons have been exposed to an infected person, thereby artificially increasing the precision of the estimates of vaccine efficacy.

Our results point to strategies to efficiently allocate catch-up vaccination efforts in heterogeneously vaccinated populations. No additional vaccination is needed in schools with high vaccination coverage (>75%, say) as these are already protected against epidemic outbreaks affecting a large fraction of students. Similarly, allocating vaccines to schools with low vaccination coverage (<50%, say) is inefficient as it does not markedly reduce the probability of infection for those who are not vaccinated, i.e. the indirect benefits of vaccination are small in these populations. Our analyses suggest that vaccination of populations in the range between these two extremes is most efficient, and that in these populations a single vaccination can potentially prevent almost two infections. Of course, in practice other considerations, for instance on ethical issues, communication, and cost-effectiveness would also come into play.

## Methods

### Study design and data collection

In the Netherlands, several large outbreaks of mumps virus (genotype D) occurred in 2007–2009. We collected data from children attending primary schools with evidence of mumps virus transmission (report of at least one laboratory confirmed mumps case or more than one clinical mumps case) [9], [11]. Children's parents were asked to fill out a questionnaire asking for information on the child's vaccination status and occurrence of mumps. Individual data on vaccination status were also retrieved from the national Dutch vaccination register. When these were not available, we used the self-reported vaccination status (vaccinated/unvaccinated). Children who were vaccinated more than twice (one case), and who were reported to have had mumps before September 2007 (three cases) were excluded. The study was approved by the medical ethics committee of the University Medical Centre Utrecht and the Radboud University Nijmegen Medical Centre. The data are presented in Table 1 and Tables S1, S2.

### Estimation of vaccine efficacy in a disease transmission framework

#### Model structure

The analyses are based on the distribution of the number of persons infected in an outbreak [16]–[18]. Specifically, we use so-called final size distributions of a two-type SEIR (susceptible-exposed-infectious-recovered) model in which the two types represent unvaccinated and vaccinated persons.

In SEIR models, each individual in the population can either be susceptible (i.e. healthy), exposed (infected but not able to infect others), infective (infected and able to infect others) or recovered (not infectious and now immune). We assume that infectious contacts are made at the level of the school, and not at other organizational levels (e.g., class, household, community). These assumptions seem reasonable since there was no evidence of sustained community transmission during the study period, while only limited information was available on class structure within schools.

We focus on estimation of two key epidemiological quantities, the basic reproduction number 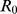 which quantifies the transmissibility of the pathogen, and vaccine efficacy 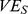 which determines the reduction in the probability of infection for those who have been vaccinated. We use a Bayesian inferential framework in which these parameters are estimated and the missing information is imputed.

Throughout we assume that a pair of individuals makes contacts at a rate that is inversely proportional to the school size *N*, thus ensuring that each person makes an identical expected number of contacts per unit of time. Specifically, whilst infective an unvaccinated person makes infectious contacts with each unvaccinated individual according to a Poisson process of rate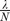, and with each vaccinated individual according to a Poisson process of rate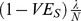, where 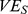 represents vaccine efficacy for susceptibility [1]. All Poisson processes are assumed mutually independent. Notice that vaccine efficacy as defined here can be interpreted as the reduction in the probability of infection for a contact that would have resulted in infection if the contacted person was unvaccinated. Hence, we have 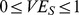 by definition.

Final size data alone do not allow us to estimate parameters with respect to calendar time, but only relative to other model parameters. To set a time-scale, and for simplicity, we therefore assume that the infectious period (i.e. the time that an individual is in the infective state) is fixed at length 1 time unit and set the basic reproduction number 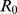
equal to the contact rate parameter 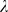. An alternative possibility, which could also be incorporated into our modelling and inference framework, is to assume that infectious periods are exponentially distributed with mean 1, which yields a geometric distribution for the number of contacts made by an individual whilst they remain infective [30], [31]. In practice, this alternative choice of infectious period distribution rarely makes any material difference to the results [18].

Our model contains two epidemiological parameters, namely the basic reproduction number 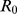 determining overall transmissibility, and vaccine efficacy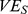 which quantifies the extent to which vaccination reduces the probability of becoming infected by a single contact. In a large population with vaccination coverage 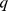 the reproduction number in the early stages of an epidemic, 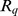, takes a simple form, namely 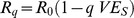 [32]. The critical vaccination coverage 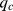 which makes major outbreaks highly improbable is found by solving the above equation for 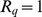, yielding
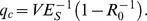
This equation is used to estimate the critical vaccination coverage directly from estimates of the basic reproduction number and vaccine efficacy.

The parameters 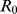, 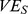, and 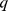 also determine outbreak size in a large population through the final size equations 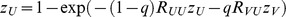
and 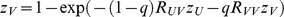, where 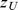 and 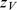 denote the fractions infected in the unvaccinated and vaccinated groups, and 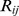 (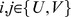) represent the type-reproduction numbers [32]. In our model we have 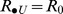 and 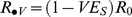.

#### The likelihood function

In a Bayesian framework the key object of interest is the posterior density of the model parameters 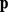 given data 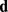, 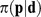. Using Bayes' rule the posterior density can be expressed as 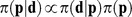, where 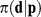 is the likelihood and 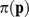 the prior density of the parameters. In practical applications, this formulation is of limited use because the likelihood 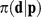 can be extremely complicated, even if no infection or vaccination information is missing. We therefore adopt an alternative approach in which attention is shifted to the joint posterior density of the parameters of interest and random directed graphs (digraphs) 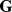 which describe the potential pathways of infection. Specifically, a given graph 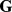
describes all directed contacts made between individuals, some of which may correspond to actual infections (e.g. a link from an infective to a susceptible) while others may not (e.g. a link from a susceptible to an infective). Knowledge of 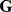, and the identity of the initially infective individual(s), determines which individuals in the population ultimately become infected, i.e. the final outcome. Full details of this method are given in [18], [19] and so we now recall the salient points in our setting.

The augmented posterior density is given by
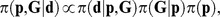
where 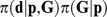 is the augmented likelihood, and 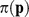 is the prior density of the parameters [18]. The (augmented) likelihood contains two factors, of which the first indicates whether a certain combination of parameters and digraphs is compatible with the data, i.e. 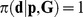 if the parameters and digraphs are compatible with the data, and 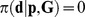 otherwise. The second factor gives the likelihood of a certain infection graph conditional on the values of the transmission parameters. For a single school the likelihood of a digraph 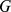 given parameters 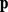, 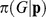, is given by the product of the likelihoods of all infectious links and non-links (i.e. the absence of an infectious link) in the set of infected and potentially infected persons, times the product of all non-links from infected persons to persons who were known to be uninfected. Hence, in a school with 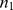 infected persons, 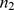 potentially infected persons, and 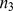 uninfected persons, 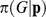 is given by the product of the probabilities of all 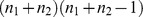 links and non-links in the set of infected and potentially infected persons, times the product of the probabilities of the non-links from persons that are infected in the digraph to persons who are known to be uninfected. Since there are at least 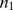 infected persons and 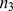 uninfected persons the number of non-links to uninfected persons is at least 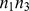. It can be higher if some of the persons with unknown infection status are infected, and reaches a maximum of 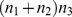 if all persons with unknown infection status are infected.

The above description can be made mathematically precise [18], [19]. If we denote by 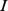 the (unobserved) set of infected individuals (of which there are at least 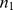 and at most 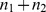), by 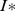 the set of individuals who are known to be infected together with the individuals who may or may not have been infected, by 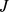 the set of individuals who are known to be uninfected, by 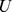 (for unvaccinated) and 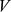 (for vaccinated) the possible person types, by 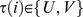 the type of an individual with label 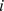, by 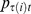 the probability that there is a link from the individual with label 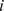 to an individual of type 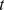, by 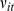 the number of links from individual 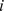 to persons of type 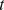, by 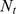 the number of individuals of type 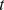 in 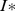 (
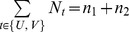
), and by 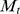 the number of individuals of type 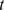 in 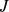 (
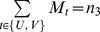
), then the likelihood 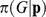 is given by
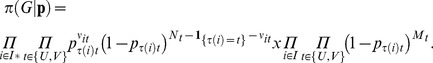
(1)Recall that we assume that the infectious periods are of fixed duration. Together with our earlier assumption that contacts are made according to mutually independent Poisson processes with rates that are inversely proportional to school size, the infection probabilities 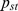 (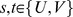) are given by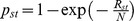, where 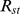 are the type-specific reproduction numbers, Our parameterization implies 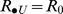 and 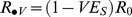.

#### Scenarios and estimation

The parameter vector 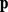 contains the epidemiological parameters 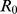 and 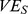, and the unknown vaccination statuses. Throughout, the basic reproduction number and vaccine efficacy are assigned uninformative uniform prior distributions (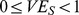 and 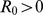). We further assume that the probability that a person with unknown vaccination status is vaccinated is given by the observed vaccination coverage of the school in which the person resides (Table 2). We consider two scenarios: One in which both 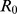 and 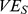 are identical across schools, and the other in which 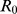 and 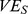 are estimated for each school separately.

The posterior density is explored using a Markov chain Monte Carlo method, whereby the missing vaccination statuses are included as latent parameters [18]–[20]. Digraphs are updated by adding and deleting edges at random from 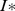. Specifically, we use a birth-death construction for updating, in which infectious contacts between any two persons that are (potentially) infected are chosen uniformly at random [18]–[20]. The Metropolis-Hastings acceptance probability of adding an infectious link to a digraph 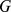 resulting in a digraph 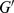, is given by 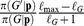, where 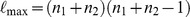 is the maximum number of infectious contacts, and 
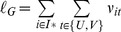
 the number of infectious contacts in 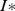. Likewise the acceptance probability of an attempt to delete an edge is 
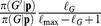
, where it is understood that digraphs that are not compatible with the data have zero likelihood. Notice that, in contrast to earlier studies, the likelihood ratio of two graphs differing by one link in general does not reduce to the likelihood ratio of having an infectious contact at a particular position versus not having an infectious contact at that position [18]–[20]. In particular, it is possible that by adding or deleting an infectious contact the number of infected persons increases or decreases by more than one, because an addition or deletion of an infectious contact could result in the addition or deletion of a number of vertices to the connected component which determines the final size. Hence, updating of the graphs requires calculation of the full likelihood of the proposed graph, which is a computationally expensive operation, resulting in long runtimes if the number of infected persons is large.

The basic reproduction number and vaccine efficacy are updated with a random-walk Metropolis algorithm using Gaussian proposal distributions with standard deviations of 0.2–2 and 0.02–0.2, respectively. Vaccination statuses are updated by flipping the vaccination status of a randomly selected person with unknown vaccination status [20]. The index case is assumed to be an unvaccinated person.

Updating is performed in blocks, in the order 1) update the value of the reproduction number, 2) update the value of vaccine efficacy, 3) for each school update the vaccination status of a randomly chosen person with missing vaccination information, 4) for each school attempt to add an infectious contact, and 5) for each school attempt to delete an infectious contact. Each cycle of the chain thus contains 32 updating events. To improve mixing every 50^th^ cycle the positions in the digraph of infected persons (both vaccinated and vaccinated) are randomly permuted so that the chain does not get stuck in topologies from which links to persons with unknown infection status cannot easily be removed. Notice that this operation leaves the topology of the graph intact (distribution of links and types), and thus does not affect the likelihood.

After running a number of exploratory analyses output is generated for a single chain of length 30,000–50,000, of which the last 20,000–25,000 cycles are used to obtain a thinned sample of size 5,000 or 10,000. Inspection of convergence of the chain is performed visually. Run times are approximately 7–10 days on a 3.2Ghz eight-core workstation.

### Simulated outbreaks

To explore the correspondence between the parameter estimates with the data, we simulated outbreaks in schools of size 200 using the digraph construction described above. To prevent early extinction we introduced three infectious persons with random vaccination status in each simulation. For each vaccination composition, we generated 5,000 random digraphs with the values of the basic reproduction number and vaccine efficacy sampled without replacement from the posterior distribution. Subsequently, for each graph we calculated the attack rate among those that were initially susceptible, and present the median and 2.5% and 97.5% percentiles of the resulting distributions (the black line and grey area in Figure 2).

### Estimation of vaccine efficacy by the cohort method

To compare our results with estimates of vaccine efficacy using the cohort method [14], we have calculated vaccine efficacy as 1 minus the relative risk of infection in vaccinated versus unvaccinated persons. In these analyses only information of persons with known vaccination and infection status was taken into account (Table S1). As in the above we employ a Bayesian framework in which the probabilities in the unvaccinated and vaccinated groups are assigned uniform prior distributions, yielding beta-binomial posterior distributions for the infection probabilities. Estimates are obtained using Markov chain Monte Carlo (MCMC) methods, specifically by taking a thinned sample of 10,000 from a converged chain of length 500,000. Table S3 reports classical (frequentist) estimates of vaccine efficacy using the cohort method [14].

## Supporting Information

Table S1 Overview of the outbreaks of mumps in Dutch primary schools. See Ruijs et al. (2011) (ref [9]) and Snijders et al. (2012)(ref [11]) for details.
(DOC)

Table S2 Summary statistics of the study population, distinguishing between one and two vaccinations (cf. Table 1). The column ‘number infected’ shows the possible range of actual infections, ranging from the number known to be infected to the sum of this number and the number of persons with unknown infection status. Vaccination coverage and attack rates are calculated using persons with known vaccination status (vaccination coverage), and known vaccination and infection status (attack rates).
(DOC)

Table S3 Classical estimates of vaccine efficacy by the cohort method, i.e. as 1 minus the relative risk of infection in vaccinated versus unvaccinated persons (Orenstein et al. 1985) (ref [14]). Approximate 95% confidence intervals of the parameter estimates are given between brackets.
(DOC)
