# Supplementary material for: Estimation of Vaccine Efficacy and Critical Vaccination Coverage in Partially Observed Outbreaks
Source: PLoS Comput Biol. 2013 May 2;9(5):e1003061. doi: 10.1371/journal.pcbi.1003061 (PMC3642050; doi:10.1371/journal.pcbi.1003061)
Supplement: Table S1 — Overview of the outbreaks of mumps in Dutch primary schools. See Ruijs et al. (2011) (ref [9]) and Snijders et al. (2012)(ref [11]) for details. (DOC) [file pcbi.1003061.s001.doc]

(Table S1 continued)

| school 6 |  | | |  |
| --- | --- | --- | --- | --- |
|  | unvaccinated | vaccinated | unknown | total |
| infected | 19 | 9 | 0 | 28 |
| not infected | 8 | 84 | 0 | 92 |
| unknown | 4 | 6 | 133 | 143 |
| total | 31 | 99 | 133 | 263 |
| school 7 |  | | |  |
|  | unvaccinated | vaccinated | unknown | total |
| infected | 6 | 0 | 0 | 6 |
| not infected | 25 | 126 | 0 | 151 |
| unknown | 5 | 2 | 30 | 37 |
| total | 36 | 128 | 30 | 194 |
| school 8 |  | | |  |
|  | unvaccinated | vaccinated | unknown | total |
| infected | 2 | 1 | 0 | 3 |
| not infected | 39 | 161 | 0 | 200 |
| unknown | 3 | 0 | 21 | 24 |
| total | 44 | 162 | 21 | 227 |
| school 9 |  | | |  |
|  | unvaccinated | vaccinated | unknown | total |
| infected | 2 | 4 | 0 | 6 |
| not infected | 9 | 130 | 0 | 139 |
| unknown | 0 | 3 | 110 | 113 |
| total | 11 | 137 | 110 | 258 |
| school 10 |  | | |  |
|  | unvaccinated | vaccinated | unknown | total |
| infected | 3 | 3 | 0 | 6 |
| not infected | 7 | 139 | 0 | 146 |
| unknown | 0 | 0 | 56 | 56 |
| total | 10 | 142 | 56 | 208 |

Table S1. Overview of the outbreaks of mumps in Dutch primary schools. See Snijders et al. (2012) and Ruijs et al. (2011) for details.
