# Supplementary material for: Estimation of Vaccine Efficacy and Critical Vaccination Coverage in Partially Observed Outbreaks
Source: PLoS Comput Biol. 2013 May 2;9(5):e1003061. doi: 10.1371/journal.pcbi.1003061 (PMC3642050; doi:10.1371/journal.pcbi.1003061)
Supplement: Table S2 — Summary statistics of the study population, distinguishing between one and two vaccinations (cf. Table 1). The column ‘number infected’ shows the possible range of actual infections, ranging from the number known to be infected to the sum of this number and the number of persons with unknown infection status. Vaccination coverage and attack rates are calculated using persons with known vaccination status (vaccination coverage), and known vaccination and infection status (attack rates). (DOC) [file pcbi.1003061.s002.doc]

|  | number of persons | number infected | vaccination coverage | attack rate  (no vaccination) | attack rate (one vaccination) | attack rate  (two vaccinations) |
| --- | --- | --- | --- | --- | --- | --- |
| all schools | 2493 | 510-1342 | 0.62^*^ | 0.68 (485/709) | 0.03 (15/582) | 0.03 (10/370) |
| school 1 | 432 | 205-369 | 0.12 | 0.86 (204/237) | 0 (0/20) | 0.09 (1/11) |
| school 2 | 338 | 135-289 | 0.13 | 0.82 (131/160) | 0.15 (3/20) | 0.25 (1/4) |
| school 3 | 259 | 68-159 | 0.42 | 0.72 (68/94) | 0 (0/47) | 0 (0/27) |
| school 4 | 184 | 40-70 | 0.54 | 0.53 (37/70) | 0.02 (1/51) | 0.06 (2/33) |
| school 5 | 130 | 13-33 | 0.75 | 0.46 (13/28) | 0 (0/52) | 0 (0/30) |
| school 6 | 263 | 28-171 | 0.76 | 0.70 (19/27) | 0.11 (6/55) | 0.08 (3/38) |
| school 7 | 194 | 6-43 | 0.78 | 0.19 (6/31) | 0 (0/82) | 0 (0/44) |
| school 8 | 227 | 3-27 | 0.79 | 0.05 (2/41) | 0.01 (1/100) | 0 (0/62) |
| school 9 | 258 | 6-119 | 0.93 | 0.18 (2/11) | 0.03 (2/75) | 0.03 (2/59) |
| school 10 | 208 | 6-62 | 0.93 | 0.30 (3/10) | 0.03 (2/80) | 0.02 (1/62) |

Table S2. Summary statistics of the study population, distinguishing between one and two vaccinations (cf. Table 1). The column ‘number infected’ shows the possible range of actual infections, ranging from the number known to be infected to the sum of this number and the number of persons with unknown infection status. Vaccination coverage and attack rates are calculated using persons with known vaccination status (vaccination coverage), and known vaccination and infection status (attack rates).

^*^: averaged over schools
