# Supplementary material for: Estimation of Vaccine Efficacy and Critical Vaccination Coverage in Partially Observed Outbreaks
Source: PLoS Comput Biol. 2013 May 2;9(5):e1003061. doi: 10.1371/journal.pcbi.1003061 (PMC3642050; doi:10.1371/journal.pcbi.1003061)
Supplement: Table S3 — Classical estimates of vaccine efficacy by the cohort method, i.e. as 1 minus the relative risk of infection in vaccinated versus unvaccinated persons (Orenstein et al. 1985) (ref [14]). Approximate 95% confidence intervals of the parameter estimates are given between brackets. (DOC) [file pcbi.1003061.s003.doc]

|  | vaccine efficacy ($1-\hat{R}R$) |
| --- | --- |
| school 1 | 0.96 (0.74 - 0.99) |
| school 2 | 0.80 (0.50 - 0.92) |
| school 3 | 1 |
| school 4 | 0.93 (0.79 - 0.98) |
| school 5 | 1 |
| school 6 | 0.86 (0.73 - 0.93) |
| school 7 | 1 |
| school 8 | 0.87 (-0.36 – 0.99) |
| school 9 | 0.84 (0.20-0.97) |
| school 10 | 0.93 (0.69-0.98) |

Table S3. Classical estimates of vaccine efficacy by the cohort method, i.e. as 1 minus the relative risk of infection in vaccinated versus unvaccinated persons (Orenstein et al. 1985). Approximate 95% confidence intervals of the parameter estimates are given between brackets.
